# Comparing the efficacy of a novel mucoadhesive patch containing *Nigella sativa* 10% with triamcinolone 0.1% in patients with erosive‐atrophic oral lichen planus: A pilot study

**DOI:** 10.1002/cre2.886

**Published:** 2024-05-26

**Authors:** Atessa Pakfetrat, Zahra Delavarian, Mahshid Malakooti, Hossein Bagheri, Habibollah Esmaily, Mahsa Ghorbani, Pooya Saeedi

**Affiliations:** ^1^ Oral and Maxillofacial Diseases Research Center Mashhad University of Medical Sciences Mashhad Iran; ^2^ Department of Oral Medicine, School of Dentistry Mashhad University of Medical Science Mashhad Iran; ^3^ Dental Materials Research Center Mashhad University of Medical Sciences Mashhad Iran; ^4^ Department of Epidemiology and Biostatistics Mashhad University of Medical Sciences Mashhad Iran; ^5^ School of Dentistry Mashhad University of Medical Sciences Mashhad Iran

**Keywords:** mucoadhesive patch, *Nigella sativa*, oral lichen planus, triamcinolone

## Abstract

**Objective:**

This study evaluates the efficacy of a novel mucoadhesive patch containing *Nigella sativa* 10% extract compared to triamcinolone 0.1% in alleviating symptoms and reducing lesion severity in patients with erosive‐atrophic oral lichen planus.

**Methods and Materials:**

A pilot study comprising two groups, each with 10 patients, was conducted. The intervention group received mucoadhesive patches containing *N. sativa* 10% extract, while the control group received triamcinolone acetonide 0.1% patches. Pain and burning intensity, measured through visual analog scale, and lesion severity based on the Thongprasom scale were assessed weekly for 4 weeks. Descriptive records were kept for side effects and patient satisfaction.

**Results:**

Pain and burning intensity decreased in both groups throughout the sessions, with the *N. sativa* group showing a greater reduction than the triamcinolone group. The reduction in burning intensity within each group was significant (*p* < .001), and there was a significant difference between groups only in the second session (*p* = .045). The overall difference between groups was not significant (*p* > .05). Lesion severity also decreased significantly in both groups (*p* < .001), with a significant difference between groups observed in the third session (*p* = .043) and overall throughout the study (*p* = .006).

**Conclusion:**

The use of *N. sativa* extract in mucoadhesive patches was as effective as corticosteroids in reducing pain, burning, and lesion severity in patients with oral lichen planus, with *N. sativa* showing superior results in some sessions. Notably, no significant complications were observed with *N. sativa* use, making it a promising treatment option for lichen planus.

## 1 INTRODUCTION

Oral lichen planus (OLP) is a chronic inflammatory disorder affecting the oral mucosa, characterized by painful erosions, ulcers, and atrophy. OLP's prevalence varies between 0.5% and 2% in different populations, with oral lesions in 50%–60% and skin lesions in 10%–15% of cases. The condition predominantly affects individuals over 40 years, with a female‐to‐male ratio of 3:2, rarely occurring in children (Alajbeg et al., 2021). The treatment of OLP often involves the use of corticosteroids, which can be associated with various side effects, including hyperglycemia, hypertension, and increased susceptibility to fungal infections (Coondoo et al., 2014; Dhar et al., 2014). As a result, there is a growing interest in exploring alternative treatments with herbal and natural origins, aiming to reduce the reliance on conventional drugs and their adverse effects while providing long‐term efficacy.

*Nigella sativa* has gained considerable attention due to its numerous therapeutic properties. It exhibits anti‐inflammatory, antitumoral, and antioxidant effects and antifungal property which are mechanisms that play a significant role in inhibiting the pathogenesis of OLP (Assi et al., 2016). By reducing inflammatory cytokines, inhibiting the cyclooxygenase pathway, and decreasing free radicals, *N. sativa* extract shows potential in treating OLP. Additionally, its antifungal properties eliminate the need for concurrent antifungal treatment, offering a more convenient approach. Although limited studies have explored the impact of *N. sativa* extract on oral ulcers, the available evidence suggests its beneficial role in improving oral ulceration (Al‐Attass et al., 2016). These findings suggest that *N. sativa* extract could serve as an effective aid in the treatment of OLP, potentially reducing the associated side effects of chemical drugs.

One of the challenges in utilizing topical corticosteroids for oral cavity treatment is the limited adhesion of the drug to the mucosa. Traditional ointments are water repellent and require the mucosal surface to be dried before application. Moreover, their adhesion to the mucosa is often inadequate, resulting in the rapid washing away of the ointment. In contrast, mucoadhesive formulations have shown promise in adhering to the mucosal surfaces. These formulations, often macromolecules with hydrophilic properties, possess functional groups capable of forming hydrogen bonds. By interacting with mucin, the major component of mucus, mucoadhesive materials establish adhesion and enhance drug delivery (Punitha & Girish, 2010). For the first time in this research, a mucosal patch of *N. sativa* has been developed, introducing a targeted drug delivery system that provides advantages such as precise localized drug dosage and enhanced bioavailability (Boddupalli et al., 2010).

In light of the known side effects of corticosteroids and the limited studies on the potential benefits of *N. sativa* extract in managing oral ulcers like chemotherapy‐induced oral mucositis (Hussain et al., 2019) and oral ulcers associated with Behcet's disease (Abdali, 2009), there is a need to compare the therapeutic effects of mucoadhesive formulations containing *N. sativa* extract with triamcinolone, a commonly used corticosteroid.

This pilot study aimed to assess the effectiveness of two types of mucoadhesive patches, namely *N. sativa* and triamcinolone, in reducing pain and burning sensation, as well as their effects on improvement of oral lesions.

## 2 MATERIALS AND METHODS

### 2.1 Study design

The primary goal of this balanced nonrandomized [1:1], parallel‐designed pilot study was to compare the efficacy of *N. sativa* 10% with triamcinolone (RAHA Pharmaceutical Co.) mucoadhesive patches in treating patients diagnosed with erosive‐atrophic OLP.

The clinical procedures were performed at the Oral and Maxillofacial Diseases Department, Mashhad Dental School. The laboratory procedures, including preparing *N. sativa* 10% extract and mucoadhesive patches, were conducted at the School of Pharmacy and Dental Materials Research Laboratory, Mashhad University of Medical Sciences, Mashhad, Iran, between October 2020 and August 2021. The main objectives of this study were to measure the effectiveness of the treatments by examining the proportion of patients who showed improvements in pain levels and the severity of their oral lesions over the course of 4 weeks. Pain reduction was assessed using the visual analog scale (VAS), while the severity of the lesions was evaluated based on the Thongprasom scoring system. These measurements were recorded at weekly intervals throughout the study duration.

Before the commencement of interventions, all participants were required to provide informed consent, which was approved by the Vice‐Chancellor of Research at Mashhad University of Medical Sciences. Detailed information regarding the study's objectives and methods was provided to the participants, and any questions they had were addressed. The study was granted ethical approval by the Ethics Committee of Mashhad University of Medical Sciences, with the ethical code IR.MUMS.DENTISTRY.REC.1397.098, thereby ensuring the utmost protection of patient confidentiality. The research was conducted in adherence to the principles outlined in the Declaration of Helsinki.

### 2.2 Formulation of therapeutic patches

#### 2.2.1 Preparation of the mucoadhesive patches

The mucoadhesive patches were prepared using a two‐layer structure. The first layer, serving as the base mucoadhesive, consisted of chitosan (Sigma‐Aldrich), gelatin (Suvchem), and glycerin (Neutron Co.) in weight percentages of 30, 60, and 10, respectively, based on a previous thesis (Bagheri, 2021). To create the first layer, chitosan powder was added to 17.5 molars of 1% acetic acid solution, followed by mixing (Alfa D‐500 mixer) at 90°C and 20 rpm for 1 h to form a homogeneous 1% chitosan solution. Gelatin powder was then added to distilled water to create a 5% gelatin solution, which was mixed until homogeneous. The solutions of chitosan, gelatin, and glycerin were combined according to specified ratios and spread onto a petri dish to a thickness of 0.2 mm. The first layer was left to dry at room temperature for 24–48 h.

Once the first layer had dried, the second layer was prepared to enhance adhesion to the mucosa and facilitate drug delivery. The second layer consisted of a mixture of pectin (Sigma‐Aldrich), carboxymethyl cellulose (CMC) (Sigma‐Aldrich), and gelatin in varying ratios. A 5% solution of CMC in water was prepared and mixed to achieve uniformity. Similarly, 5% solutions of gelatin and pectin were prepared separately. The solutions were then combined, and either triamcinolone (0.1%) or *N. sativa* extract (10 mg/g) was added to the mixture. By carefully preparing and assembling the two layers with their respective compositions, the final mucoadhesive patches were created for use in the study.

#### 2.2.2 Preparation of *N. sativa* extract and triamcinolone powder

The extraction of *N. sativa* was carried out by a pharmacologist and a specialist in traditional medicine. *N. sativa* seeds were purchased from Traditional Medicine Health Center, Mashhad, Iran and finely ground using a grinder. The ground material was then placed inside a filter paper and subjected to extraction using the Soxhlet extraction method. A 75% hydroalcoholic solution, consisting of 75% alcohol and 25% water, was utilized as the solvent for the extraction process. After 24 h, the resulting liquid extract was separated from the solvent through the use of an apparatus, yielding pure *N. sativa* extract. Approximately, 6.8 g of pure extract were obtained from every 100 g of *N. sativa*. Triamcinolone powders were also purchased from a pharmaceutical company (RAHA Pharmaceutical Co.).

#### 2.2.3 Quality and characteristics assessment

The quality assessment of the patches in our study followed the methodology outlined in the previously conducted thesis (Bagheri, 2021). After the preparation of suitable samples, the quality and characteristics of the mucoadhesive patch containing either *N. sativa* or triamcinolone were evaluated. Several assessments were undertaken, encompassing a moisture absorption test (Delta Incubator), solubility evaluation, pH meter (Multi 9420, WTW), mucosal adhesion assessment, punch test, and tensile strength measurement (all three were performed using Santam STM‐20 model), as well as drug release analysis (Franz Diffusion Cell, Gallenkamp thermostirrer 100, Germany). Based on the results, the optimal formulation with regard to mechanical strength, adhesion, and drug release capability was selected.

### 2.3 Clinical procedures

Twenty adult patients diagnosed with erosive‐atrophic OLP required treatment with a mean age of 53.4 ± 12.2 years were recruited from Mashhad Dental School referral patients. Table 1 provides comprehensive inclusion and exclusion criteria. The eligibility was verified by the principal investigator.

**Table 1:** Inclusion and exclusion criteria.

Inclusion criteria	Exclusion criteria
New cases of erosive‐atrophic OLP confirmed both clinically and histopathologically	Patients with special medical conditions classified as ASA III and higher
Patients with OLP who have not received any treatment for a duration of 2 weeks or more due to various reasons	Presence of lesions in posterior and inaccessible areas of the mouth
Limited to accessible mucosal areas, with involvement restricted to a maximum of two areas	Histopathological results indicating lichenoid reaction, moderate to severe dysplasia
Maximum diameter of 3 cm	Documented allergic reaction to *Nigella stavia*
Minimum score of 2 on the Thongprasom criteria	Pregnant and lactating women
Patient consent to participate in the study	Children under 8 years of age
	Recent use of immunosuppressive and anti‐inflammatory medications within the past month

Personal information, including age, gender, medical history, medication use, and smoking history, was recorded in the relevant forms. The oral cavities of eligible patients were examined by an oral and maxillofacial disease specialist, using the conventional oral examination (COE) (Essat et al., 2022). The location, size of the lesions (cm^2^), clinical description, and accompanying symptoms were documented. Pain and burning sensation levels were assessed by the same clinician, using the VAS (Table 2). Lesion size was measured with a caliper, and severity was classified according to the Thongprasom grading system (Table 3).

**Table 2:** Severity of pain according to visual analog scale (VAS).

Severity	VAS score
Extremely severe	7.5–10
Severe	5–7.4
Moderate	2.5–4.9
Mild	0–2.4

**Table 3:** Thongprasom scoring system.

Severity	Thongprasom score
White striae with erosive area ≥ 1 cm^2^	5
White striae with erosive area < 1 cm^2^	4
White striae with atrophic area ≥ 1 cm^2^	3
White striae with atrophic area < 1 cm^2^	2
White striae without erythematous areas	1
Absence of lesions (normal mucosa)	0

The patients were divided into two groups, each consisting of 10 individuals. Both groups received similar and clear instructions regarding the application of the respective products. The intervention and control groups used mucoadhesive patches with *N. sativa* 10% extract and triamcinolone 0.1%, respectively. The patches were to be applied three times a day, with an 8‐h interval left in place for at least 30 min without touching or consuming anything. After the designated time, if the patch remained attached, the area was moistened, and the mouth was rinsed. Both groups were also prescribed Nystatin 100,000 units/mL drop (Emad Pharmaceutical Co.) to prevent fungal infections. Patients were advised to use the drop three times a day, with 20–30 drops per session, ensuring a 2‐h interval between its use and the application of the patch. Each mouth rinse session should last 3–5 min, followed by spitting out the mouthwash. Patients refrained from consuming anything for at least 30 min after rinsing.

Patients underwent regular follow‐up sessions throughout the study, scheduled at T1 (first week), T2 (second week), T3 (third week), and T4 (fourth week). These sessions monitored patients' progress, evaluated pain, burning sensation, lesion characteristics, and recorded data for analysis and comparison. The evaluations were performed using the same methodology employed during the baseline assessment (T0). Additionally, any observed side effects and the patients' satisfaction with the treatment were also recorded. Patients were instructed not to initiate new medications without informing the investigators. Following the completion of the study, follow‐up care was provided by the Department of Oral and Maxillofacial Diseases, independent of the current study's scope. It is essential to note that after the initial 4‐week period, patients with faded lesions were scheduled for follow‐up sessions, while patients with smaller lesions that had not faded underwent more frequent weekly follow‐up sessions until complete resolution and then scheduled for follow‐up sessions.

### 2.4 Statistical analysis

To ensure adequate statistical power and reliability of the study results, the sample size was determined based on various factors including the research objectives, available resources, and feasibility considerations. Considering the pilot nature of the study and the absence of similar studies on the therapeutic intervention with mucoadhesive patches, a sample size of 20 participants was deemed appropriate. The data were presented using frequency tables, line graphs, and mean values with standard deviation. Statistical analysis involved the application of different tests, such as Fisher's exact test, independent samples *t*‐test, and repeated measures analysis of variance (ANOVA). A significance level of *p* < .05 was considered to determine statistical significance. The statistical analysis was performed using SPSS software (version 26; SPSS Inc.). Additionally, a 95% confidence interval was calculated for the study results.

## 3 RESULTS

**Figure 1:**
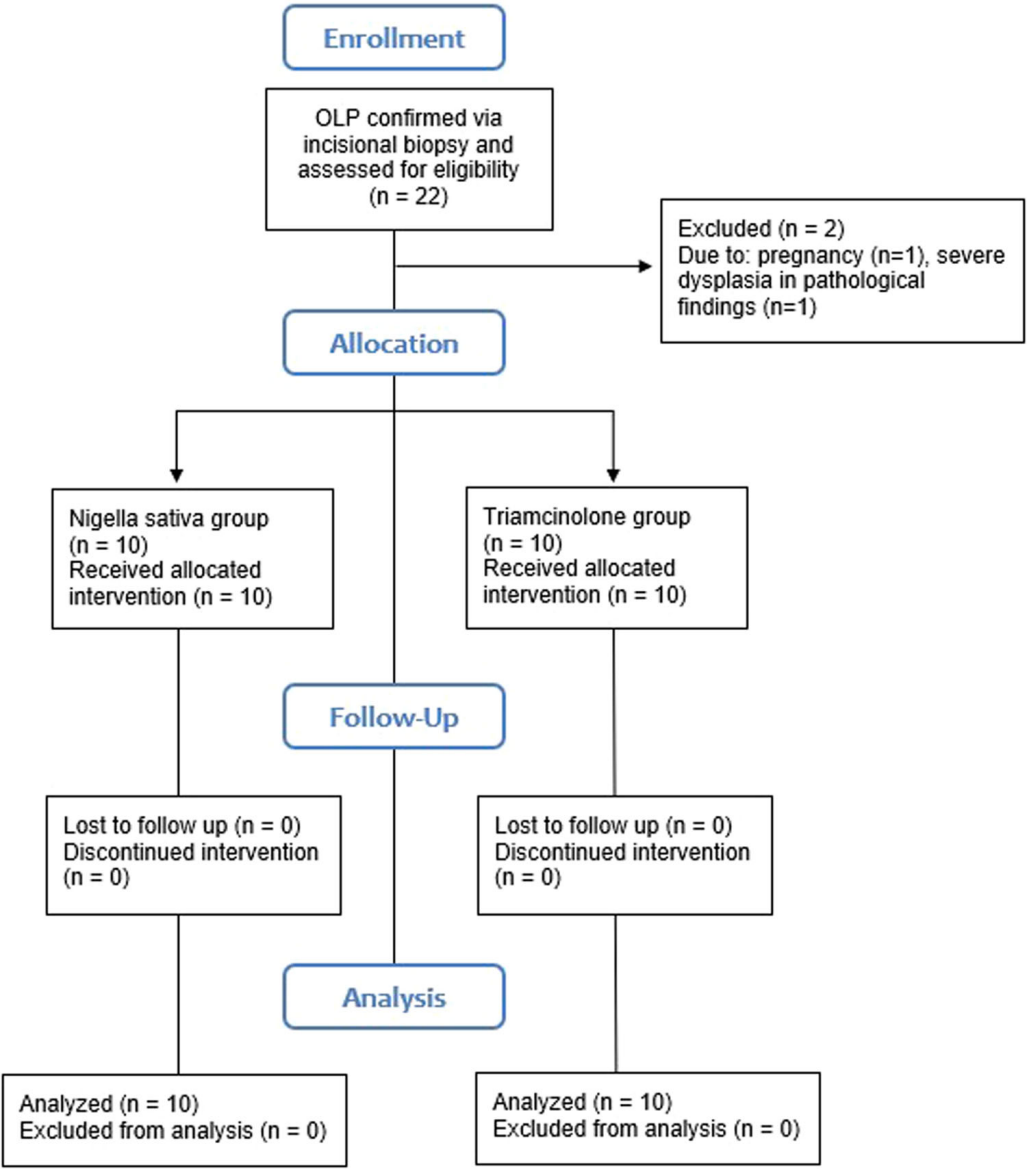
CONSORT diagram showing the flow of patients through the trial.

Figure 1 illustrates the patient flow through the trial. Twenty patients were enrolled in the trial, with a mean age of 53.4 ± 12.2 years and a gender ratio of 13 females (65%) to 7 males (35%). No significant differences were observed in terms of gender (*p* = 1.000) and age (*p* = .223) between the groups, as indicated by Fisher's exact test and the independent t‐test, respectively. Mainly, the buccal mucosa showed the highest frequency of lesions, with 12 out of 17 lesions in the *N. sativa* group and 10 out of 16 lesions in the triamcinolone group. Notably, some participants had multiple lesions within their oral cavity, which contributed to an elevated lesion count compared to the number of participants. Nevertheless, the distribution of lesion locations between the groups showed no significant difference, as indicated by Fisher's exact test (*p* = .879). Additionally, the distribution of systemic conditions (*p* = .881) and smoking history (*p* = 1.000) between the groups were analyzed using the independent t‐test and Fisher's exact test, respectively, and found to be insignificant. The distribution of OLP‐associated factors, such as cutaneous symptoms (*p* = 1.000), OLP history duration (*p* = .141), and histopathological dysplasia (*p* = 1.000), were assessed using Fisher's exact test and found to be statistically insignificant. Table 4 presents the distribution of baseline characteristics in both groups. Furthermore, Table 4 illustrates the pretreatment distribution of lesion severity based on the Thongprasom criterion in both groups. Notably, grade 3 lesions exhibited the highest frequency in terms of severity, whereas grade 2 lesions had the lowest frequency.

**Table 4:** Baseline data distribution in intervention and control groups.

Baseline variables				*Nigella sativa*	Triamcinolone	Total	*p*‐Value
Gender	Male		*N*	3	4	7	1.000^a^
			%	30	40	35	
	Female		*N*	7	6	13	
			%	70	60	65	
Age	(mean ± SD)			56.8 ± 13.0	50.0 ± 10.9	53.4 ± 12.2	.223^b^
Lesion location	Buccal mucosa		*N*	12	10	22	.879^a^
	Gingiva		*N*	2	3	5	
	Tongue		*N*	3	3	6	
Systemic disease	Hypertension		*N*	4	3	7	.881^b^
	Hypothyroidism		*N*	2	2	4	
	Absence of systemic disease		*N*	4	5	9	
Smoking habits	Yes		*N*	3	2	5	1.000^a^
	No		*N*	7	8	15	
OLP‐associated conditions	Cutaneous symptoms	Yes	*N*	2	1	3	1.000^a^
		No	*N*	8	9	17	
	OLP history	>1 yr	*N*	5	1	6	.141^a^
		<1 yr	*N*	5	9	14	
	Dysplasia	Mild	*N*	0	1	1	1.000^a^
		No	*N*	10	9	19	
Lesion severity based on Thongprasom	Grade 2		*N*	2	2	4	
	Grade 3		*N*	7	6	13	
	Grade 4		*N*	6	5	11	
	Grade 5		*N*	2	3	5	

*Note*: A 95% confidence interval of the mean was estimated in the analysis.

^a^ The *p* values are the results of Fisher's exact test.

^b^ The *p* values are the results of independent *t*‐tests.

Before being evaluated, participants in two groups of 10 received either *N. sativa* 10% or triamcinolone 0.1% mucoadhesive patches. The assessment was conducted using the VAS and Thongprasom scoring system at five following time points: before treatment (T0), 1 week (T1), 2 weeks (T2), 3 weeks (T3), and 4 weeks (T4) after treatment.

**Figure 2:**
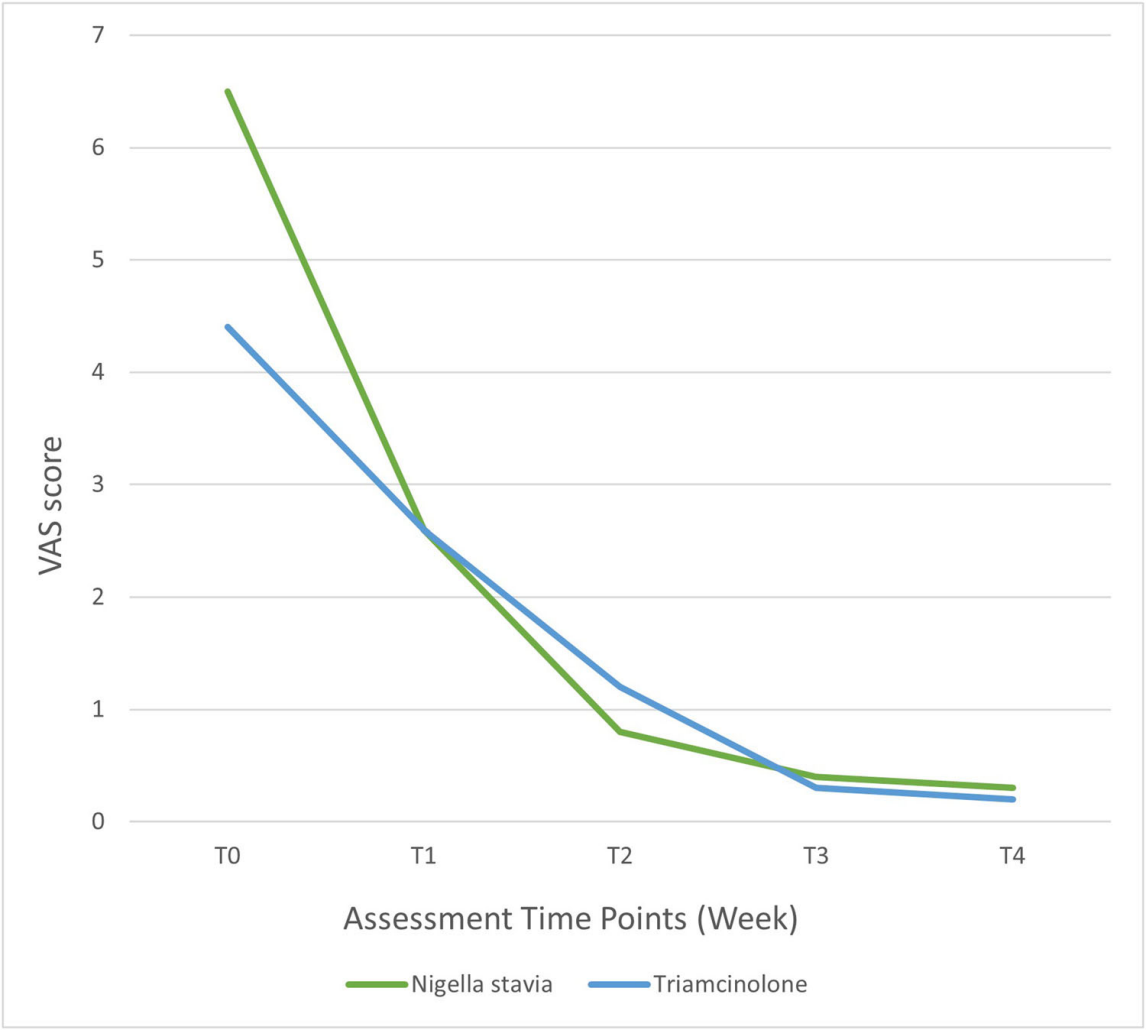
Line graph illustrating the mean pain and burning sensation according to the visual analog scale (VAS) criterion within the two groups during treatment sessions.

**Figure 3:**
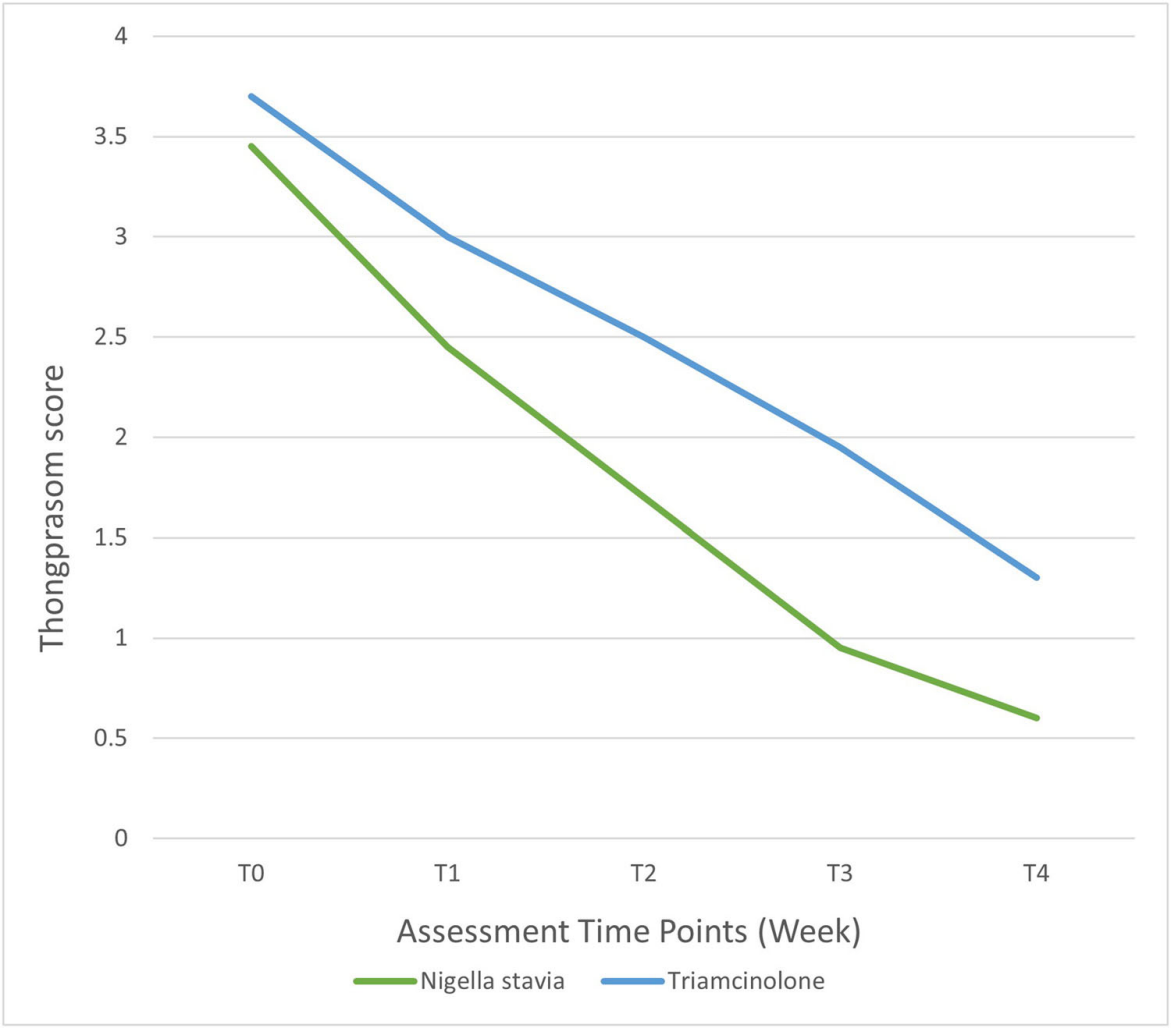
Line graph illustrating the mean severity of lesions based on the Thongprasom criterion within the two groups during treatment sessions.

As illustrated in Figures 2 and 3, the consistent downward trajectory in reducing pain and burning sensation and lesion severity throughout multiple weeks for both groups is evident. This trend prompted the evaluation of the decrease in VAS and Thongprasom scores for each session relative to the baseline.

Referring to Table 5, as confirmed by the independent *t*‐test, the initial mean VAS and Thongprasom scores displayed no significant difference between the two study groups (*p* = .516 for both). This reaffirmed their similarity at the baseline. Moreover, over examination sessions, both groups exhibited diminishing VAS and Thongprasom scores. Notably, the *N. sativa* group achieved a more pronounced overall reduction compared to the triamcinolone group.

**Table 5:** Mean pain and burning sensation (VAS) and lesion severity (Thongprasom) before and during treatment sessions in both study groups.

Variables		*Nigella stavia*	Triamcinolone	*p*‐Value^a^
		Mean ± SD	Mean ± SD	
VAS	T0	6.50 ± 2.59	4.40 ± 3.33	.516
	T0–T1	3.90 ± 2.51	1.80 ± 1.87	.050
	T0–T2	5.70 ± 2.58	3.20 ± 2.61	.045*
	T0–T3	6.10 ± 2.64	4.10 ± 3.21	.146
	T0–T4	6.20 ± 2.69	4.20 ± 3.15	.145
	*p*‐Value^b^	<.001*	<.001*	
Thongprasom	T0	3.45 ± 0.72	3.70 ± 0.94	.516
	T0–T1	1.00 ± 0.91	0.70 ± 0.58	.395
	T0–T2	1.75 ± 0.82	1.20 ± 0.53	.097
	T0–T3	2.50 ± 0.78	1.75 ± 0.75	.043*
	T0–T4	2.85 ± 0.78	2.40 ± 0.80	.223
	*p*‐Value^b^	<.001*	<.001*	

*Note*: A 95% confidence interval of the mean was estimated in the analysis.

* Indicates a significant difference (*p* < .05).

^a^ The *p*‐values are the results of independent *t*‐tests.

^b^ The *p*‐values are the results of repeated measures analysis.

The independent *t*‐test analysis unveiled no significant differences in VAS score reduction during the first, third, and fourth weeks between the groups (*p* = .050, *p* = .146, *p* = .145, respectively). Conversely, during the second week, the intervention group demonstrated a significant reduction in VAS score (*p* = .045). Concerning Thongprasom scores, the independent t‐test outcomes indicated no significant differences in lesion severity reduction between the groups during the first, second, and fourth weeks (*p* = .395, *p* = .097, *p* = .223, respectively). However, during the third week, a significant reduction was noted, with the *N. sativa* group exhibiting a more pronounced decline in lesion severity (*p* = .043).

Furthermore, based on repeated measures analysis, the reduction in both variables demonstrated statistical significance over the 4‐week period in both *N. sativa* and triamcinolone groups, yielding P values of <0.001 for both variables in both groups.

The repeated measures analysis in Table 6 demonstrated that, even when adjusting for age and gender, there was no significant difference in pain and burning sensation levels within each group. Age and gender did not impact changes in VAS scores (*p* = .147 and *p* = .205, respectively) nor did the type of study group (*p* = .408). Notably, a pattern emerged: significant reduction in VAS scores during the first and second weeks compared to the final week (*p* = .001 and *p* = .018, respectively), while the third week did not exhibit this significant reduction (*p* = .331).

**Table 6:** Impact of variables on pain and burning sensation (VAS) and lesion severity (Thongprasom) reduction during the trial.

			Model coefficient	Standard error	*T*	*p*‐Value
VAS	Groups	*Nigella stavia*	0.373	0.43	0.8	.408
		Triamcinolone	0			
	Time points	T1	2.400	0.57	1.4	.001*
		T2	1.000	0.38	2.6	.018*
		T3	0.100	0.10	0.1	.331
		T4	0			
	Gender	Female	0.547	0.41	1.3	.205
		Male	0			
	Age		0.025	0.01	1.5	.147
Thongprasom	Groups	*Nigella stavia*	0.905	0.29	3.0	.006*
		Triamcinolone	0			
	Time points	T1	2.400	0.25	9.5	<.001*
		T2	1.700	0.24	6.8	<.001*
		T3	1.200	0.22	5.2	<.001*
		T4	0			
	Gender	Female	0.077	0.25	0.3	.764
		Male	0			
	Age		0.029	0.01	2.7	.014*

*Note*: The *p*‐values are the results of repeated measures analysis. A 95% confidence interval of the mean was estimated in the analysis.

* Indicates a significant difference (*p* < .05).

In terms of lesion severity, accounting for gender showed no significant difference in the change of Thongprasom scores within the two groups (*p* = .764). However, when age was factored in, a significant difference emerged, indicating a correlation between age and the reduction in lesion severity (*p* = .014). Notably, a substantial difference was observed between the *N. sativa* and triamcinolone groups in terms of Thongprasom scores reduction (*p* = .006), favoring a significantly more substantial reduction in the *N. sativa* group. Across the first, second, and third weeks, there was a significant reduction in Thongprasom scores compared to the final week (*p* < .001 for all).

No harmful effects were noted during the course of the trial. However, a minority of participants in the intervention groups reported certain complaints, such as experiencing a bitter taste and expressing a preference for single‐dose medication. Additionally, a few complaints, including challenges in drug administration and mouth burning, were reported in both groups by a limited number of individuals. Importantly, none of the study participants were excluded due to these reported complaints.

## 4 DISCUSSION

This study aimed to assess the comparative effectiveness of *N. sativa* mucoadhesive patch and triamcinolone mucoadhesive patch in alleviating pain and burning sensations based on VAS, as well as evaluating the degree of improvement in oral lesion severity using Thongprasom in patients diagnosed with erosive‐atrophic OLP through biopsy confirmation. Following eligibility checks, a total of 20 patients were recruited from referrals of Mashhad Dental School to participate in this 4‐week trial.

As per the data analysis, pain and burning sensation intensity consistently decreased in both groups throughout the trial. The *N. sativa* group exhibited a notably greater overall reduction than the triamcinolone group, with significant differences only in the second week. Likewise, lesion severity consistently decreased in both groups during examination sessions, with a more notable reduction in the *N. sativa* group, particularly significant in the third week.

The prescription of *N. sativa* mucoadhesive patch demonstrated comparable efficacy to the triamcinolone mucoadhesive patch in reducing pain, burning sensation, and lesion severity. This was evidenced by a notable reduction in inflammation and lesion improvement after a 4‐week duration, signifying its equivalence to the conventional corticosteroid treatment. Moreover, these innovative specialized patches introduce a novel approach that release their active ingredients gradually upon adhering to the mucosal surface, resulting in quicker, localized effects and enhanced bioavailability due to bypassing liver metabolism and digestive enzyme breakdown. Furthermore, these patches can effectively shield oral lesions from painful and burning stimuli, preventing heightened irritation.

The results indicated the potential benefits of the suggested mucoadhesive patch for patients diagnosed with erosive‐atrophic OLP, where involvement is limited to a maximum of two areas and a maximum diameter of 3 centimeters. Nevertheless, given the study's limited sample size, it's advisable to encompass a diverse range of OLP types, including severe cases, and a wider age group in future trials to validate the treatment's effectiveness against the entire spectrum of the disease.

Apart from a thesis by Sarabadani et al. (2014), clinical trial studies examining the effects of *N. sativa* on OLP lesions have been scarce. Therefore, making a direct comparison between our current study and recent research might pose some challenges. In Sarabadani et al.'s (2014) thesis, 100% *N. sativa* oil mouthwash was prescribed and administered three times a day for 4 weeks. Similar to our approach, their study employed the VAS criteria to gauge patients’ pain and burning sensation and used the Thongprasom criteria to assess lesion severity before treatment and throughout the first, second, third, and fourth weeks of therapy. Their conclusion indicated the absence of a significant difference between the intervention and control groups, which could potentially be attributed to the use of corticosteroids in both groups. Furthermore, their findings also pointed toward the necessity for further investigations to comprehensively understand the potential impact of *N. sativa* on OLP lesions. Noteworthy side effects, such as burning sensations, nausea, complaints about the oil's texture, and bitterness, were observed in the intervention group. This prompted us to reduce the concentration to 10%, resulting in decreased bitterness while retaining effectiveness. Moreover, transitioning from oil application to the mucoadhesive patch eliminated the discomfort associated with oil use.

Nonetheless, certain studies have explored the efficacy of *N. sativa* in addressing different types of oral lesions. Given its capacity to accelerate the healing process and its favorable impact on oral ulcer recuperation, we can draw certain parallels when assessing its potential effects on the healing and improvement of erosive‐atrophic OLP ulcers. A clinical trial by Hussain et al. (2019) aimed to compare *N. sativa* mouthwash with the conventional “magic mouthwash” (nystatin, tetracycline, lidocaine, and dexamethasone) for chemotherapy‐induced oral mucositis in patients with acute myeloid leukemia. The study demonstrated improved outcomes, including decreased mucositis severity, pain relief, and enhanced swallowing function. This investigation was primarily motivated by *N. sativa*'s compound benefits, particularly thymoquinone, which possesses wound‐healing, analgesic, anti‐inflammatory, and antimicrobial properties. These effects align with its potential to reduce inflammation, epithelial damage, and pain in OLP lesions. In Abdali's study (2009), the goal was to investigate the effects of *N. sativa* oil on oral‐genital ulcers associated with Behcet's disease. One group received topical treatment with a glycerin‐*N. sativa* oil combination, applied three times daily, while the control group received glycerin alone. Assessments were conducted on Days 4 and 8 after treatment. The results strongly indicated a significant therapeutic impact of the oil in improving Behcet's disease‐related ulcers and effectively alleviating pain, which aligns with our findings of enhancing OLP lesions. Notably, the study highlighted the absence of toxicity or side effects.

The central objective of this research remained the investigation of *N. sativa*'s potent anti‐inflammatory effects, with a special emphasis on its key component, thymoquinone.

Many therapeutic effects of *N. sativa*, such as antioxidant, antimicrobial, anti‐inflammatory, immune‐modulating, analgesic, antiarthritic, antidiabetic, antiasthmatic, and anti‐eoplastic properties, have been attributed to thymoquinone, a key compound present in *N. sativa*.

Some animal and in vitro studies have directly extracted thymoquinone from *N. sativa* and yielded promising results, emphasizing its potential in various therapeutic applications (Kotowski et al., 2017; Nasuti et al., 2019). As previously mentioned, the prolonged administration of corticosteroids for managing OLP, along with epithelial thinning of the lesions, increases susceptibility to Candida fungal colonization. This underscores the requirement to incorporate antifungal agents like nystatin into the treatment protocol. Thymoquinone, as indicated by diverse laboratory investigations (Gawron et al., 2019; Goel & Mishra, 2018; Kouidhi et al., 2011; Mouwakeh et al., 2019; Randhawa et al., 2015), exhibits both antifungal and antibacterial attributes, thus eliminating the need for supplementary antifungal medications. Moreover, some animal investigations have explored the histopathology of wound healing involving *N. sativa*, revealing encouraging findings (Al‐Douri & Al‐kazaz, 2010; Çanakci et al., 2018). However, as the clinical confirmation of the antifungal properties of *N. sativa* is pending, the authors opted for ethical considerations and prescribed nystatin for both groups.

The study's limitations included a restricted sample size and a relatively short follow‐up period, attributed to COVID‐19‐related restrictions. While our preliminary results displayed promise, conducting additional research with a larger participant pool and an extended observation duration would enhance our understanding of the prolonged impacts and viability of the *N. sativa* mucoadhesive patch. Additionally, the absence of blinding and randomization is another aspect to acknowledge. For forthcoming clinical trials, implementing blinding and randomization methodologies would validate and strengthen the credibility of our findings. Furthermore, including a control group in future studies would be advantageous for a comprehensive evaluation of the effectiveness of substances in the patches. This is important as the patch itself, without any medication, may potentially improve outcomes simply by physically covering the lesions.

The study presents several strengths, including the pioneering use of the *N. sativa* mucoadhesive patch as an adhesive wound dressing. Additionally, the inclusion of nanotechnology in this innovative patch facilitated accurate drug delivery to the oral mucosa, marking the novel introduction of this drug delivery method. Moreover, the study's incorporation of *N. sativa*, a readily available, indigenous, and cost‐effective herbal remedy with antifungal properties, provides a chemical‐free substitute for nystatin‐based treatment. In future clinical studies, it would be valuable to explore the effectiveness of thymoquinone as an active ingredient, alongside investigating different concentrations of *N. sativa*. Considering the well‐established antifungal properties of *N. sativa*, upcoming clinical trials should prioritize examining this particular aspect.

## 5 CONCLUSION

The study revealed that *N. sativa* effectively reduces pain and burning sensation and lesion severity similar to corticosteroids, with some instances showing better results for *N. sativa*. Notably, no significant adverse effects were observed. Both types of mucoadhesive patches containing *N. sativa* and triamcinolone were successfully explored for drug delivery. In conclusion, the *N. sativa* mucoadhesive patch holds promise as a successful treatment for OLP based on promising results.

## AUTHOR CONTRIBUTIONS

**Atessa Pakfetrat**: Conceptualization; formal analysis; methodology; project administration; supervision; writing—review and editing. **Zahra Delavarian**: Conceptualization; methodology; supervision; writing—review and editing. **Mahshid Malakooti**: Visualization; Investigation; Methodology; writing—review and editing. **Hossein Bagheri**: Methodology; writing—review and editing. **Habibollah Esmaily**: Formal analysis; writing—review and editing. **Mahsa Ghorbani**: Investigation; visualization; writing—original draft; writing—review and editing. **Pooya Saeedi**: Investigation; visualization; writing—original draft; writing—review and editing.

## CONFLICT OF INTEREST STATEMENT

The authors declare no conflict of interest.

## ETHICS STATEMENT

The study was granted ethical approval by the Ethics Committee of Mashhad University of Medical Sciences, with the ethical code IR.MUMS.DENTISTRY.REC.1397.098, thereby ensuring the utmost protection of patient confidentiality. The research was conducted in adherence to the principles outlined in the Declaration of Helsinki.

## Data Availability

The data that support the findings of this study are available on request from the corresponding author.
